# Interpretations of education about gene-environment influences on health in rural Ethiopia: the context of a neglected tropical disease

**DOI:** 10.1093/inthealth/ihw016

**Published:** 2016-07-30

**Authors:** Abebayehu Tora, Desta Ayode, Getnet Tadele, David Farrell, Gail Davey, Colleen M. McBride

**Affiliations:** a Addis Ababa University, College of Social Sciences, P.O. Box 180505, Addis Ababa, Ethiopia; b People Designs Inc., 1304 Broad St, Durham NC, 27705, USA; c Wellcome Trust Centre for Global Health Research, Brighton and Sussex Medical School, Falmer, Brighton BN19PX, UK; d Emory Rollins School of Public Health, 1518 Clifton Rd NE, GCR 564, Atlanta GA 30322, USA

**Keywords:** Ethiopia, Gene-environment, Health education, Podoconiosis

## Abstract

**Background:**

Misunderstandings of the role of genetics in disease development are associated with stigmatizing behaviors and fatalistic attitudes about prevention. This report describes an evaluation of community understanding of an educational module about genetic and environmental influences on the development of podoconiosis, a neglected tropical disease endemic in highland Ethiopia.

**Methods:**

A qualitative process assessment was conducted as part of a large prospective intervention trial in August 2013, in Wolaita Zone, southern Ethiopia. Sixty five participants were purposively selected from 600 households randomized to receive the inherited susceptibility module. The educational module used pictorial representations and oral explanations of the interaction of inherited sensitivity and soil exposure and was delivered by lay health educators in participants' homes. Data were collected using semi-structured individual interviews (IDIs) or focus group discussions (FGDs).

**Results:**

Qualitative analyses showed that most participants improved their understanding of inherited soil sensitivity and susceptibility to podoconiosis. Participants linked their new understanding to decreased stigma-related attitudes. The module also corrected misconceptions that the condition was contagious, again diminishing stigmatizing attitudes. Lastly, these improvements in understanding increased the perceived value of foot protection.

**Conclusions:**

Taken together, these improvements support the acceptability, feasibility and potential benefits of implementing gene-environment education in low and middle income countries.

## Introduction

By all accounts genetic discovery and technology development have been progressing at an ever-quickening pace.^1^ Leaders around the world have raised concerns that these trends may mean that health benefits arising from genetic discovery will not reach, or perhaps even be relevant for, the low and middle income countries (LMICs) that could stand to benefit most.^2–4^ Indeed, WHO's Human Genetics Programme has outlined a number of goals aimed to offset such disparity, including the need to, ‘build public understanding of the science of human genetics and genomics, related technologies and health services; and their ethical, legal and social implications’.^5^

It is increasingly clear that most human disease pathogenesis is the product of complex interaction of multiple genes along with environmental and behavioral risk factors, now commonly referred to as genomics.^6,7^ However, conveying the complexity of gene by environment interactions to lay audiences when they have limited genetic literacy is very challenging.^8, 9^ There is widespread concern and some evidence in LMICs to support that the lay public tends not to appreciate this complexity and to be deterministic in thinking that genetic diseases are unavoidable.^10–12^ In turn, it has been documented that beliefs about the inevitability of genetic diseases have the potential to negatively impact health-seeking, preventive health behaviors and interpersonal interactions.^13–15^

Engaging target populations within LMICs to understand nuances and in turn persuade them that health conditions can be avoided also will be challenging.^16–19^Among the most important challenges are the low literacy levels that characterize LMICs and the limited public health infrastructures available to support educational interventions.^20^

Podoconiosis offers a disease context in which to evaluate genetics education efforts. Podoconiosis is a neglected tropical disease that occurs in tropical Africa, Central America and north India;^21^ the average prevalence within Ethiopia is 4%.^22^ Evidence to date suggests that walking barefoot in soil with high levels of silica particles leads to lymphatic inflammation among genetically susceptible families.^23,24^ Although podoconiosis is known to cluster within families in endemic areas, community members lack understanding of the joint role of environmental and hereditary influences.^11^

A local nongovernment organization, Mossy Foot International (MFI), has been conducting health education regarding podoconiosis prevention for over a decade in endemic areas of southern Ethiopia. MFI had previously avoided discussion of heredity as a contributor to podoconiosis based on concerns that such discussions could exacerbate interpersonal stigma directed to affected families. Instead their educational efforts emphasized the benefits of foot hygiene and use of proper footwear, efforts known to reduce disease risk. However, our prior qualitative work indicated that a sizeable number of community members, particularly unaffected families, continued to hold beliefs that heredity was a contributor, beliefs that continued to justify decisions not to marry into affected families and other stigmatizing behaviors.^11^

We conducted a cluster randomized intervention trial between January 2013 and June 2014 that is described in detail elsewhere.^25^ Briefly, trial results indicated that levels of accurate knowledge and reported stigma improved in communities that received a lay health educator (LHE)-delivered educational module and 3-month follow-up booster sessions about inherited susceptibility and prevention of podoconiosis. These trends were largely observed for the families in unaffected households, with less intervention benefit observed among affected households.^25^

This report describes a qualitative process assessment conducted with the 600 households that were randomized to receive the inherited susceptibility educational module. The aim for this supplemental data collection was to explore participants' understanding of the module and to indirectly assess the quality of the LHEs' household sessions.

## Materials and methods

### LHEs training on inherited susceptibility module

Twenty LHEs were selected to deliver household skills training sessions as described elsewhere.^25^ LHEs were coached using role play, group exercises and individual presentations to master understanding of concepts and to develop confidence, and to deliver the core messages in the module. Additionally, LHEs were trained in communication and listening skills. Pre- and post-test assessments were conducted to assess LHEs' mastery of these skills.

### Inherited susceptibility education module

The inherited susceptibility education module (ISEM) was delivered orally by LHEs during a home visit with affected and unaffected families. Our rationale for evaluating the ISEM module with unaffected households is that inherited susceptibility may be a factor among those without a positive family history of disease. Additionally, individuals may have incomplete knowledge of their family history when relatives have moved away or have died. Genetic research is incomplete in the context of podoconiosis making it difficult to know who carries inherited susceptibility. Lastly, high levels of stigma can limit willingness of individuals to report family history. The objectives of the ISEM were therefore to increase participants' understanding that while podoconiosis is hereditary it is entirely preventable; present a locally meaningful metaphor to explain how inherited or genetic ‘susceptibility’ paired with environmental factors increases risk for podoconiosis; link this explanation to the special importance of foot protection among those who are susceptible; and correct misperceptions concerning contagiousness and other folk understandings of podoconiosis risk (Table 1). Training guides were developed to convey this information that included a variety of materials to supplement LHE's oral explanations such as case presentations, story books, and pictures.

**Table 1.** Summary of inherited susceptibility module

Concept	Messages	Examples/supplemental materials
Heredity	Heredity means that traits get passed down from one generation to another Some inherited traits that pass from parents to children are unavoidable and unchangeable (height, eye color) Not all traits are on this ON-OFF switch.	Using appearance characteristics that ‘run in families’ as a way to clarify the definition. Showing photos of families with varying appearance.
Inherited sensitivity	Individuals inherit differences in their sensitivity to the environment Individuals can accommodate these sensitivities by changing their behavior. Sun sensitivity is an example In the case of podoconiosis, it is soil sensitivity. An individual's sensitivity to the soil is an inherited characteristic and is not contagious	Graphically represented sun sensitivity metaphor
Environmental exposure	Silica particles in the soil are thought to cause the feet to swell for those with soil sensitivity	Exercise to view soil and see silica particles
Inherited susceptibility + exposure	Individuals with inherited soil sensitivity are most affected by silica particles in soil. Walking barefoot is most harmful for these individuals. Proper foot hygiene and wearing shoes is most beneficial. All individuals can benefit from wearing shoes for foot health	Graphical people depicting the joint effects of soil sensitivity and soil exposure to increase risk and benefit of foot protection.

**Figure 1.**
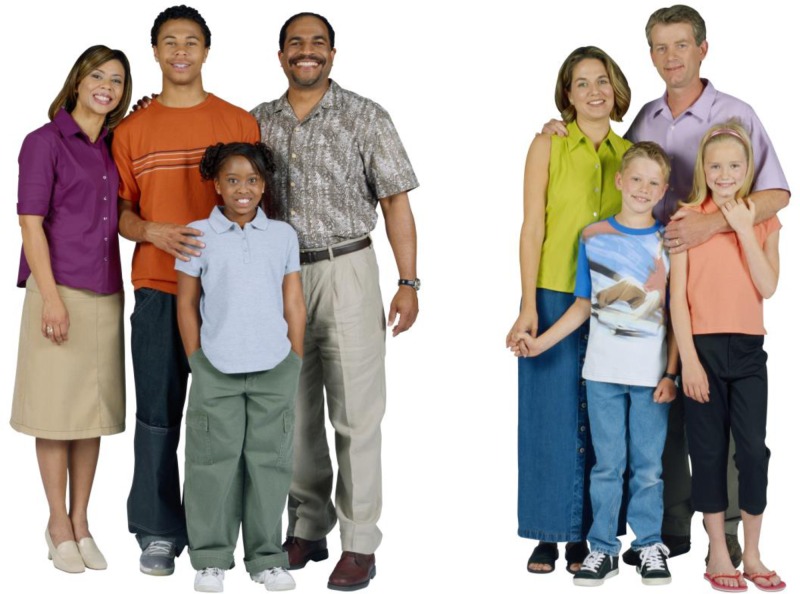
Family photos describing non-controllable hereditary attributes. This figure is available in black and white in print and in color at International Health online.

For the purpose of simplification, in the materials and LHE training, we used family photos (see Figure 1) to demonstrate how inherited traits of physical make-up such as skin and hair color are passed down from parents to offspring. In the case of podoconiosis, the ISEM discussed the distinction between inheriting physical traits and inheriting susceptibility to help participants understand that individuals with affected relatives may inherit ‘soil sensitivity traits’, not the disease itself. The messages framed to communicate the conceptual distinction between ‘inheriting susceptibility traits’ and ‘inheriting disease itself’ have been summarized in Table 1.

**Figure 2.**
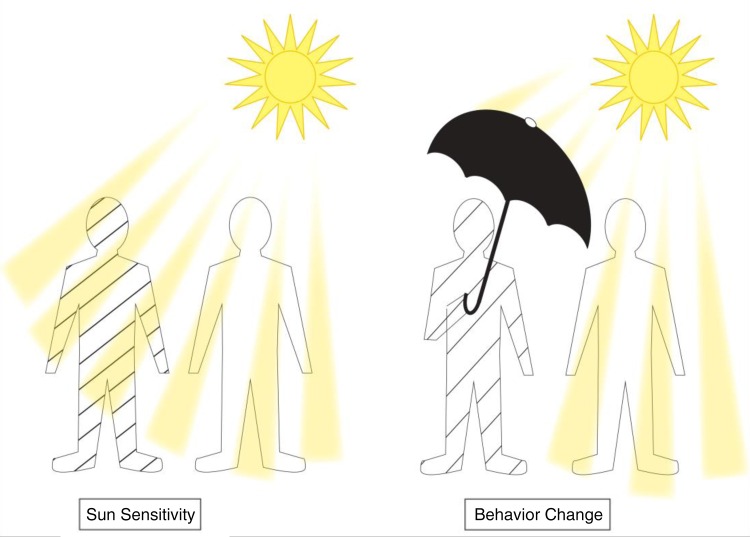
Sun sensitivity metaphor demonstrating benefit of adopting preventive action. This figure is available in black and white in print and in color at International Health online.

In order to convey the concept of inherited susceptibility, we relied on a metaphor based on sensitivity to sun exposure. One sees considerable variability in how individuals in these communities of highland Ethiopia deal with long distance walking in unavoidable sun exposure. Some carry umbrellas, others wear hats and others walk without protection. This points to individual variation in levels of tolerance to sun exposure. Qualitative pretesting showed that there were not negative cultural or value judgments regarding sun sensitivity. The ISEM likened variability in sun sensitivity to soil sensitivity and used visual images to convey this metaphor (see Figure 2). Graphical cartoons of individuals with sun sensitivity donning umbrellas were explained as an example of taking preventive actions in response to the sun even though it did not modify their inherited trait of sun sensitivity. In turn, this was likened to the importance of shoe wearing among individuals with soil sensitivity.

**Figure 3.**
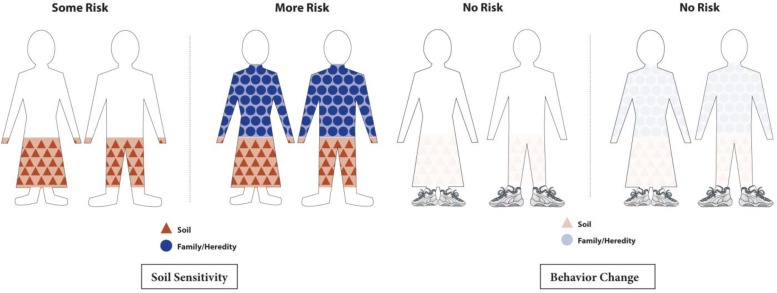
Graphical figures to convey variation in level of susceptibility and importance of wearing shoes. This figure is available in black and white in print and in color at International Health online.

Pictures of protective (closed) and non-protective (open) shoes were used to talk about the importance of maximizing protection by wearing closed toed shoes. Cartoon figures (both susceptible and not susceptible) were presented to illustrate how variable sensitivity interacts with soil exposure to increase the risk of developing podoconiosis (see Figure 3). The images showed that while everyone is exposed to the soil, wearing closed toed shoes is most important for those with inherited soil sensitivity.

Lastly, the training materials described the mechanism through which the soil is thought to be harmful to those with soil sensitivity. A mineral in the soil, namely aluminum silicate, was described as the key exposure. This information was used to bring home the message that podoconiosis is not a contagious disease. Participants were instructed by LHEs to look at the red clay soil of the area to see the shiny particles that were thought to be the culprit.

### Deployment and supervision of LHEs

LHEs visited the homes of the 600 trial participants in arm 3, a cluster of affected and unaffected households randomized to receive the ISEM. It took on average 2 hours to deliver the household education. Field managers evaluated communication skills, use of education materials and completeness and accuracy of messages delivered. In the early sessions, LHEs had trouble using training materials, skipped sessions, gave shallow presentation of major concepts in the module such as heredity, delivered incomplete messages such as saying ‘podoconiosis is hereditary’ without conveying susceptibility concepts properly, used non-participatory approaches such as one way communication, talked too fast, and did not use reflective listening techniques.

In addition to giving feedback on areas for improvement, field managers paired best performing LHEs with low performers to encourage peer mentoring and quality improvement. A supervision template was developed to record the challenges reported by LHEs and the questions that were raised during the household session. Field managers met with LHEs once a week to discuss issues raised in the supervision template and their field experiences.

### Qualitative process assessment

The qualitative process assessment was conducted in two Mossy Foot International (MFI) sites randomized to receive inherited susceptibility module. The MFI has been operating in Wolaita Zone, southern Ethiopia for over a decade. Details about selection of trial sites were described in our previous article.^25^ The qualitative assessment was carried out in August 2013 after two weeks of initial household skills training activities. A series of semi-structured in-depth individual interviews (IDI) and focus group discussions (FGDs) were conducted with a sample of 65 adults from the 600 households (200 affected, 400 neighboring unaffected households) that were randomized to receive the inherited susceptibility educational module. Thirty two individuals took part in the IDIs and 33 individuals took part in FGDs (two with affected; two with unaffected participants). Each of the FGDs had 10–13 participants. As with the overall trial, most participants in the process evaluation were female. Most interviews and all FGDs were held in the local language, Wolaitattuwa. On average, IDIs and FGDs lasted for 45 minutes and 2 hours, respectively. All the data were recorded using digital recorders, once permission was given.

Interviews were transcribed and translated into English. Identification of themes and sub themes was guided both by grounded theory approach and predefined themes in the interview guides. NVivo-10 software (NVivo, QSR International, Burlington, MA, USA) was used for qualitative data analysis along with manual coding.

## Results

The themes we focused on for this report were aimed to bring further clarity to the results of the randomized controlled trial.^25^ We evaluated the intervention's influence on unaffected and affected participants' understanding of heritability, views on stigmatizing behaviors and attitudes towards preventive behaviors.

### Participants' understanding of inherited susceptibility to soil sensitivity

The terms used for ‘heredity’ in the local language were ‘Zariyappe laatettiyaba’ referring to ‘traits inherited from generation to generation among blood relatives’. We used the local term ‘eeshsha’ as equivalent for ‘traits’. The local language phrase used in the educational module to describe ‘inherited susceptibility to sensitivity’ was ‘bolla lanchisiya eeshsha laattiyoga’. These terms were mentioned consistently by both unaffected and affected participants in their descriptions of LHEs' explanations of inherited susceptibility. Participants used the term lanchiya bolla (sensitive body), and lanchiya eeshsha (sensitive trait). However, they also used the terms, shugo bolla (soft body), dandayena bolla (less resistant body), and hanqettiya bolla (angry body).

*For instance, if the father is affected in a given family, his feet might have been swollen because he had ‘shugo bolla’* [soft body]. *His children might be soft bodied and may get the disease if they walk barefoot* (unaffected female, 35 years).

*If those people with ‘hanqettiya bolla’* [angry body] *to the soil walk barefoot, they may get the disease* (affected male, 28 years).

Many participants were able to make the conceptual distinction between inheriting disease and inheriting susceptibility, clearly describing that relatives of the affected person inherit the susceptibility, not the disease itself as illustrated in the participant's comments below.

*Some people misunderstood how the disease can be inherited. They mistakenly say the disease passes from parents to children and it is inevitable. I don't believe in this idea. For example, if my hand is amputated, there is no way to give birth to an amputated child. We were told by the household educators that the disease cannot be inherited directly, but the susceptibility* (unaffected female, 50 years).

### Examples of misunderstanding and resistance to heredity as a factor in podoconiosis

Some participants also appeared to reverse their previously held belief that podoconiosis is hereditary, deciding instead that podoconiosis is not hereditary. These participants interpreted the LHE training as telling them that barefoot exposure to soil particles was the only cause. A story told by an affected woman lends insight into this misunderstanding. When asked about the cause of podoconiosis, she explained her curiosity about how she got the disease when she had no affected relatives. She knew a family where parents and their children had podoconiosis. This led her to believe that the disease was hereditary. As a result, she became extremely worried about her children. She described how her fears were allayed by what she learned from the LHEs, that podoconiosis is not hereditary.

### Participants' understanding of combined soil exposure and susceptibility increasing risk of podoconiosis

Both affected and unaffected participants frequently mentioned barefoot exposure to soil particles to be the main environmental and behavioral risk factor. They labeled soil particles in several ways: ‘aluminium silicate’, ‘minerals’, ‘silicum’, ‘shiny things’, ‘sugar like things’, ‘toxic things’, ‘poisonous things’, and so on. Participants were able to interpret how barefoot exposure to these soil particles combined with inherited susceptibility increased disease risk.

*In my understanding, when there is contact with silicum minerals in the soil and inherited body sensitivity, these two things jointly cause the disease. However, if they protect their feet from soil contact, body sensitivity alone may not bring the disease. This is how I was taught and how I understood it* (unaffected female, 50 years).

### Misunderstanding of environment and susceptibility interrelationship

For some affected participants, the mention of environment as a risk factor appeared to justify de-emphasizing the role of heredity. The comments of an affected woman illustrate this:

*Previously, we thought that the disease is hereditary which is inherited from fathers or mothers line. But now, we heard that the ‘silicum’ causes the disease. We are convinced by this information and began to keep our feet from it* (affected female, 45 years).

In contrast, among some unaffected participants, the mention of heredity as a causal factor appeared to justify overemphasis of heredity in podoconiosis etiology. These participants interpreted the inherited susceptibility module as confirming the traditionally held belief that heredity is the sole cause. They thought it was impossible to protect the children of affected parents from getting the disease regardless of shoe wearing.

*There is no doubt that children of the affected families would be at higher risk than those in unaffected, because of heredity. They are highly susceptible to the disease even when they wear shoes. Wearing shoes delays the onset of the disease, but cannot get rid of it* (unaffected female, 28 years).

### Contagion misperceptions were corrected

An important learning objective for the inherited susceptibility module was to disabuse community members of their beliefs that podoconiosis is contagious. After participating in the ISEM, some participants acknowledged that they had wrongly perceived podoconiosis to be a contagious disease.

*Before this education we had worries that the disease may be passed to other persons through sharing of clothes and shoes. They [LHEs] said the disease is not contagious. They said it affects only those with sensitive body make up* (affected female, 30 years).

### Influence of understanding inherited susceptibility on attitudes about stigma

Most participants described that the LHE sessions had lessened their concerns about being around affected individuals because the information they received had convinced them that podoconiosis was neither contagious nor inevitable if susceptible individuals regularly wore shoes and used proper foot hygiene.

*In the previous time, it was common to avoid marriage with affected families. But, no one has come into this world with swollen feet though his or her parents' feet are swollen. Because of our ignorance, we were doubtful. But, this education benefited me a lot. At this time, I am more than happy to let my daughter get married with a person from affected families. … I will not stop her from marrying. I learned that the disease is preventable* (unaffected female, 45 years).

Participants who had previously stigmatized affected people explained the change in their attitudes provoked by the household health education intervention.

*In earlier times, we had little awareness about the disease. We thought it was contagious. As a result, we have been refraining from giving to and taking from affected persons. But, after this education, we have realized that the disease is not contagious. We are happy to share anything with them* (unaffected female, 30 years).

For most affected families learning that the disease was not contagious, and was both treatable and preventable boosted their confidence to talk about the disease to others and to take part in a range of social events.

*I was so worried about what people would say when they saw my swollen feet which they knew as normal. I even went to the clinic in secret. And I never told to others about this … Now I am so confident to talk about this disease to others. I can tell them that the disease is treatable and preventable* (affected male, 28 years).

### Linking improved knowledge to children's shoe wearing

Participants indicated that prior to receiving the ISEM they had little knowledge about the consequences of walking barefoot on the soil. Based on what they learned in the LHE session, some of them reported having decided that their perceptions of shoes as luxuries for special occasions were wrong.

*We didn't have information that the disease develops from soil. I only wear shoes for special occasions. But after we learned that the disease is caused by the soil particles, I also became worried that I may develop the disease if I walk barefoot. Thus, I started to wear shoes wherever possible* (unaffected female, 24 years).

The majority of affected parents also understood that their children needed special attention as they were more likely to have inherited soil sensitivity than children in unaffected families.

*I have worries that one or two of my children may get the disease as both my husband and I are affected. I don't want to hide this. Their feet may swell in the future. I believe that I can protect their feet helping them wear shoes and keeping their hygiene* (affected female interviewee, 38 years).

Unaffected parents who had thought the disease was not preventable were also convinced that affected parents could be successful in preventing their children from developing podoconiosis through shoe wearing and hygiene.

*I think they have very soft body and their physical makeup is different. If their children start wearing shoes since their early childhood, I think the disease will not be passed to the children and cannot continue to affect their family members* (unaffected female, 28 years).

Prior to the ISEM, most unaffected parents considered their children to be at no risk since their relatives were free from the disease. They considered only contact, intermarriage and sharing shoes with affected people to be risky for their children. However, after participating in the ISEM, many recognized that their risk perceptions were poorly informed. They realized that their children should also be wearing shoes as it is difficult to detect who has inherited susceptibility until the disease manifests. They also stressed that their children should wear shoes to protect their feet from injuries and other harmful things in the soil.

*Yes, I have worries. We know even very young children who got the disease. A number of males and females who were healthy last year have swollen feet in the current year. We didn't realize that shoe wearing and hygiene prevent the disease which we learned in this education* (unaffected female, 30 years).

## Discussion

After participating in the ISEM, a majority of participants, both those affected and unaffected by podoconiosis, improved their understanding of inherited soil sensitivity and how it interacted with soil exposure to increase the risk for the susceptibility to podoconiosis. In turn, participants reported that these understandings linked directly to lessening their stigma-related attitudes. The module also corrected their misconceptions that the condition is contagious, which in turn lessened stigmatizing attitudes. Lastly, these improvements in understanding increased the perceived value of foot protection. Taken together, these improvements support the acceptability, feasibility and potential benefit of implementing gene-environment education in LMICs.

The process evaluation also supported the promise of using culturally derived metaphors for explaining concepts in genomics and health. Such ‘common sense’ methods have been proposed to increase the coherence of explanations of health risks and as a means to encourage uptake of appropriate preventive solutions.^26^ However, how these metaphors stand up in the long term and the implications for stigma to re-ignite is as yet unclear. For example, in the context of podoconiosis, interpretations that likened susceptibility to a ‘soft’ or ‘angry’ body could over time become associated with weakness or negativity and ultimately lead back to social stigma. Thus, ongoing educational efforts likely would be beneficial to identify and address these misunderstandings before they become culturally entrenched.

Additionally, LHEs' explanations of the distinction between sensitivity to the soil as the trait that gets passed down from generation to generation rather than the disease itself were not consistently understood by participants. For unaffected participants, this misunderstanding resulted in their over-emphasizing heritability. By contrast, affected participants with this misunderstanding tended to hear only that podoconiosis is not inherited but instead is purely environmentally influenced. The latter interpretation is understandable given the long affiliation of MFI with these communities and MFI's commitment to emphasizing prevention instead of the role of heredity. In turn, this may have accounted, in part, for our prior findings of lower increases in knowledge for affected households.^25^ This is also congruent with another study which found a resistance among the public to identifying genetic components in health conditions identified as environmental.^27^ Thus, such defensive responses to information about genetics should be anticipated and incorporated into programmes aimed at improving genetic literacy.

While the LHE model showed promise for increasing genetic literacy, there were weaknesses observed during the early phases of the training and household education sessions. For example, some lay educators provided superficial presentations of the concept of heredity, improper and insufficient use of supplementary examples and visual aids, and incomplete messages (either stressing only heredity or environment).

An additional limitation is that we cannot validate an association between the qualitative assessments of intervention response and actual uptake or sustainability of any improvements in the interface between inherited susceptibility and barefoot exposure. Consecutive process assessments at intervals might yield more information on accuracy of understanding and its association with sustained shoe wearing. Our intervention relied on partnering with an NGO that served affected individuals and engagement of community leaders. Obtaining funding to continue these efforts will be challenging. Initiatives of the National Institutes of Health such as the Human Heredity and Health in Africa (H3Africa) aimed to build capacity in genomic research and program implementation and could help identify approaches to build infrastructure to support genetic literacy in LMICs.^28^

### Conclusions

In conclusion, the results have future implications for efforts in genetics literacy building. Community-wide dissemination of linguistically and culturally appropriate gene-environment education using a LHE model could be an effective dissemination strategy for improving understanding of inherited conditions, discourage deterministic thinking and stigmatizing attitudes and promote health in LMICs.
